# P23 Prevalence of asymptomatic bacteriuria and associated risk factors in an adult population relevant to UK primary care: a systematic review and meta-analysis

**DOI:** 10.1093/jacamr/dlad077.027

**Published:** 2023-08-02

**Authors:** Abi Moore, Conor Coyle, Anna Seeley, Phoebe Cherrington-Walker, Maaedah Khan, Thomas Fanshawe, Koen Pouwels, Donna Lecky, Gail Hayward, Emily Cooper

**Affiliations:** Department of Primary Care Health Sciences, University of Oxford, Oxford, UK; Department of Primary Care Health Sciences, University of Oxford, Oxford, UK; Department of Primary Care Health Sciences, University of Oxford, Oxford, UK; Department of Primary Care Health Sciences, University of Oxford, Oxford, UK; Department of Primary Care Health Sciences, University of Oxford, Oxford, UK; Department of Primary Care Health Sciences, University of Oxford, Oxford, UK; Oxford Population Health, University of Oxford, Oxford, UK; UK Health Security Agency, UK; Department of Primary Care Health Sciences, University of Oxford, Oxford, UK; UK Health Security Agency, UK

## Abstract

**Background:**

The use of urine dipstick tests in people who are more likely to have asymptomatic bacteriuria (ASB) can lead to the over-diagnosis of urinary tract infection (UTI) and unnecessary antibiotic prescribing. As a result, urine dipsticks are not recommended in those over the age of 65 years, or those with a urinary catheter in UK primary care guidelines. However, due to variation in prevalence estimates for ASB and the age of some of the existing prevalence studies it is unclear whether the use of age 65 as a cut-off for dipstick testing remains appropriate.

**Objectives:**

This systematic review aims to identify and synthesize more recent evidence around ASB prevalence and other risk factors relevant to a UK community context, in order to improve how UK primary care UTI diagnostic guidance addresses asymptomatic bacteriuria.

**Methods:**

The protocol for this systematic review is registered with PROSPERO. Inclusion criteria are: study participants aged ≥18 years; prospective studies recruiting from a primary care relevant population; studies that measure ASB prevalence and risk factors; and studies from the Organization for Economic Co-operation and Development member countries. Exclusion criteria include: studies from inpatient hospital settings (including the emergency department); studies exclusively including people who are pregnant, who have urinary catheters, who are post renal transplant or who have structural urological abnormalities; animal studies; laboratory studies; studies published before 1990; and studies not written in English. Searches were conducted in CINAHL, Embase and Medline using MeSH and free text words adapted for each database including terms for bacteriuria AND asymptomatic disease. The Cochrane Collaboration Covidence platform is being used for the management of screening and assessment of studies. Two authors are independently completing screening, data extraction and quality assessments.

**Results:**

The initial search identified 3891 studies. After screening, 685 are currently undergoing full text review. Overall findings will be reported in a narrative synthesis. We will describe the studies identified, including how ASB has been defined within each of the studies. If sufficient data are available we will complete a meta-analysis. Using standard meta-analysis methods we will generate forest plots showing prevalence and 95% CIs for each study. If possible, we will estimate pooled prevalence of ASB from the reported prevalence of eligible studies, by age group and gender.

**Conclusions:**

The results of this review may highlight where new evidence around ASB prevalence and risk factors relevant to a UK primary care population is needed.

